# Temperature Drift Compensation of Fiber Optic Gyroscopes Based on an Improved Method

**DOI:** 10.3390/mi14091712

**Published:** 2023-08-31

**Authors:** Xinwang Wang, Ying Cui, Huiliang Cao

**Affiliations:** 1 School of Instrument Science and Engineering, Southeast University, Nanjing 210018, China; 2 School of Automotive and Transportation, Wuxi Institute of Technology, Wuxi 214000, China; 3 Key Laboratory of Energy Conversion and Process Measurement and Control Ministry of Education, School of Energy and Environment, Southeast University, Nanjing 210096, China; 4 ARC Research Hub for Computational Particle Technology, Department of Chemical Engineering, Monash University, Clayton, VIC 3800, Australia; 5 Key Laboratory of Instrumentation Science & Dynamic Measurement, Ministry of Education, North University of China, Taiyuan 030051, China

**Keywords:** fiber optic gyroscope (FOG), multi-scale permutation entropy complete ensemble empirical mode decomposition with adaptive noise (MPE-CEEMDAN), adaptive Kalman filter (AKF), grey wolf optimizer-least squares support vector machine (GWO-LSSVM), temperature compensation

## Abstract

This study proposes an improved multi-scale permutation entropy complete ensemble empirical mode decomposition with adaptive noise (MPE-CEEMDAN) method based on adaptive Kalman filter (AKF) and grey wolf optimizer-least squares support vector machine (GWO-LSSVM). By establishing a temperature compensation model, the gyro temperature output signal is optimized and reconstructed, and a gyro output signal is obtained with better accuracy. Firstly, MPE-CEEMDAN is used to decompose the FOG output signal into several intrinsic mode functions (IMFs); then, the IMFs signal is divided into mixed noise, temperature drift, and other noise according to different frequencies. Secondly, the AKF method is used to denoise the mixed noise. Thirdly, in order to denoise the temperature drift, the fiber gyroscope temperature compensation model is established based on GWO-LSSVM, and the signal without temperature drift is obtained. Finally, the processed mixed noise, the processed temperature drift, the processed other noise, and the signal-dominated IMFs are reconstructed to acquire the improved output signal. The experimental results show that, by using the improved method, the output of a fiber optic gyroscope (FOG) ranging from −30 °C to 60 °C decreases, and the temperature drift dramatically declines. The factor of quantization noise (Q) reduces from 6.1269 × 10^−3^ to 1.0132 × 10^−4^, the factor of bias instability (B) reduces from 1.53 × 10^−2^ to 1 × 10^−3^, and the factor of random walk of angular velocity (N) reduces from 7.8034 × 10^−4^ to 7.2110 × 10^−6^. The improved algorithm can be adopted to denoise the output signal of the FOG with higher accuracy.

## 1. Introduction

With the advancement of navigation and guidance equipment, conventional micro-electro-mechanical system (MEMS) gyroscopes cannot meet the precision requirements of the field of navigation and guidance at this stage [1,2,3,4,5]. Therefore, looking for alternatives to MEMS gyroscopes has become the key to catering to development. The FOG is an innovative sensor device for angular velocity measurement. Since its development in the 1980s, it has been widely used in the field of navigation and guidance, and its output accuracy is directly related to the performance of the inertial navigation system. In the actual use of the FOG, affected by working environment, manufacturing process, and many other factors, in addition to useful signals, the FOG signal output is also accompanied by a large amount of drift, which makes the FOG output signal submerged in a strong drift signal, limiting the use of the FOG. Therefore, how to reduce the influence of drift on the FOG gyroscope signal and to realize the effective drift reduction of the FOG output is an interesting problem in its practical application [6,7].

Up until now, numerous attempts have been made to improve the accuracy of FOGs. Yang et al. [8] proposed an improved double-factor adaptive Kalman filter (AKF) called AMA-RWE-DFAKF to denoise FOG drift signal in both static and dynamic conditions. It was found that the performance of AMA-RWE-DFAKF is competitive with RWE-AKFG and AMA-RWE-DMAKF, but superior to CKF under static conditions. Random errors like angle random walk and bias instability are reduced by 100 times based on an Allan variance analysis. In the dynamic condition, the minimum RMSE obtained by AMA-RWE-DFAKF performs better than all considered algorithms. Gao et al. [9] conducted this research mainly on the application of a novel artificial fish swarm algorithm (NAFSA) of FOG error coefficients recalibration/identification. Their results show that the NAFSA FOG error parameters recalibration method could implement a longer recalibration interval time with higher precision in some harness application environments. Wang et al. [10] introduced a new method of modeling and compensation for FOGs based on improved particle swarm optimization (PSO) and support vector machine (SVM) algorithms, and the regression accuracy of the proposed method (in the case of mean square percentage error indicators) increased by 83.81% compared to the traditional SVM. Shen et al. [11] put forward a noise reduction algorithm based on an improved empirical mode decomposition (EMD) and forward linear prediction (FLP). The results from the applications show that the method eliminates noise more effectively than the conventional EMD or FLP methods and decreases the standard deviations of the FOG outputs after denoising from 0.17 to 0.026 under sweep frequency vibration and from 0.22 to 0.024 under fixed frequency vibration. Wang [12] proposed a new model based on fusing unscented Kalman filter (UKF) with support vector regression (SVR) optimized by the adaptive beetle antennae search (ABAS) algorithm to reduce the random error of the FOG. The experiments are conducted on the measured data of the FOG to verify the superiority of the proposed model. The experimental results show that, compared with the conventional method, in terms of the compensation accuracy for random drift data, noise intensity (NI) and Durbin–Watson (DW) value of the proposed scheme are reduced and improved by 28.57% and 9.06%, respectively. Zhang [13] proposed a fusion diagnosis method in order to minimize the influence of vibrations to the greatest extent. The results showed that the proposed fusion fault diagnosis method could perform effective and robust fault diagnosis for the FOG under vibration conditions with a high diagnostic accuracy. Zhao [14] proposed a novel temperature drift compensation method of INS based on the gravitational search algorithm (GSA) tuning SVR. The experimental results verify the effectiveness of our method during different working states. And compared with the traditional polynomial fitting method, our method has better performance in the navigation experiment. The navigation accuracy increased by more than 50%. Gao [15] presented a machine learning-based method for the temperature error compensation of the FOG. Considering the root mean square error (RMSE), mean absolute value error (MAVE), and improvement factor of FOG zero-bias stability as measurement indicators, this work proposes to construct samples of sequence with temperature trend feature extraction, which effectively improves the overall accuracy of the gyroscope.

Brzostowski et al. [16,17] raised a new method to signal denoising based on EMD and sparse optimization with application to fiber optics gyroscope measurement. The experimental results demonstrated that the novel method is superior to both the EMD-HRD and EMD-SFT approaches with a higher SNR ratio. Song et al. [18,19] proposed a hybrid algorithm of an optimized local mean decomposition–kernel principal component analysis (OLMD–KPCA) method. The Allan variance analysis results indicated that the Q, N, and B reduced from 12.915 to 2.429 × 10^−1^, 1.8 × 10^−2^ to 5.061 × 10^−4^, and 3.01 × 10^−1^ to 1 × 10^−2^ based on the X axis; from 7.680 to 1.38 × 10^−1^, 1.2 × 10^−2^ to 2.647 × 10^−4^, and 1.72 × 10^−1^ to 6 × 10^−3^ based on the Y axis; and from 7.093 to 1.25 × 10^−1^, 1 × 10^−2^ to 1.549 × 10^−4^, and 1.53 × 10^−1^ to 7 × 10^−3^ based on the X axis, respectively. Zhang et al. [20] proposed a novel algorithm based on singular spectrum analysis (SSA) and augmented nonlinear differentiator (AND) to extract the useful signal from a noisy measurement of FOGs. The proposed SSA-AND algorithm has a better denoising ability compared with other advanced denoising algorithms, and the temperature drift of the FOG can be extracted effectively without signal delay. Wang et al. [21] displayed a novel denoising method based on an improved EMD and modified recursive least squares (RLS) algorithm. The results showed that the error mean was reduced by 27.01%, and the horizontal position error was reduced by 106.75 m when the INS lasted for 1000 s. Song et al. [22] described an improved AKF based on innovation and random-weighting estimation (RWE). The quantitative results revealed that the proposed algorithm is competitive for denoising IFOG signals compared with conventional KF, RWE-based gain-adjusted adaptive KF, and RWE-based moving average double-factor adaptive KF. The N factor reduces from 5.36 × 10^−3^ to 1.24 × 10^−4^, and the B factor reduces from 3.76 × 10^−2^ to 5.41 × 10^−4^. Liu et al. [23] proposed a hybrid CEEMDAN-LWT-based model and a new method requiring only 11.3% sifting iterations of the EEMD-LWT method. Meanwhile, the rate white noise, bias instability, and quantization noise buried in the FOG output signal decreased from 3.1 × 10^−3^ to 5 × 10^−4^, from 3.52 × 10^−2^ to 5.6 × 10^−3^, and from 5.412 × 10^−1^ to 2.31 × 10^−2^, respectively. Although many research scholars at this stage have conducted in-depth research on FOGs and proposed different denoising methods for FOGs, looking at these methods, the noise reduction effect, calculation time, and generality of noise reduction are all at the current stage, which still has gaps in the standard of FOGs. Therefore, a more accurate algorithm needs to be proposed to improve the accuracy of FOGs.

In this study, a novel structure and model of #1850014 FOG are established with an improved MPE-CEEMDAN method based on AKF and GWO-LSSVM, which are proposed to address the temperature drift. The output data of the X axis with the temperature ranging from −30 °C to 60 °C is discussed. Moreover, the proposed methods are compared with the Allan variance analysis method corresponding to the #001FOG performance to improve the practicability and significance of the proposed methods.

## 2. Algorithm

### 2.1. The Algorithm of MPE-CEEMDAN Method

**Figure 1:**
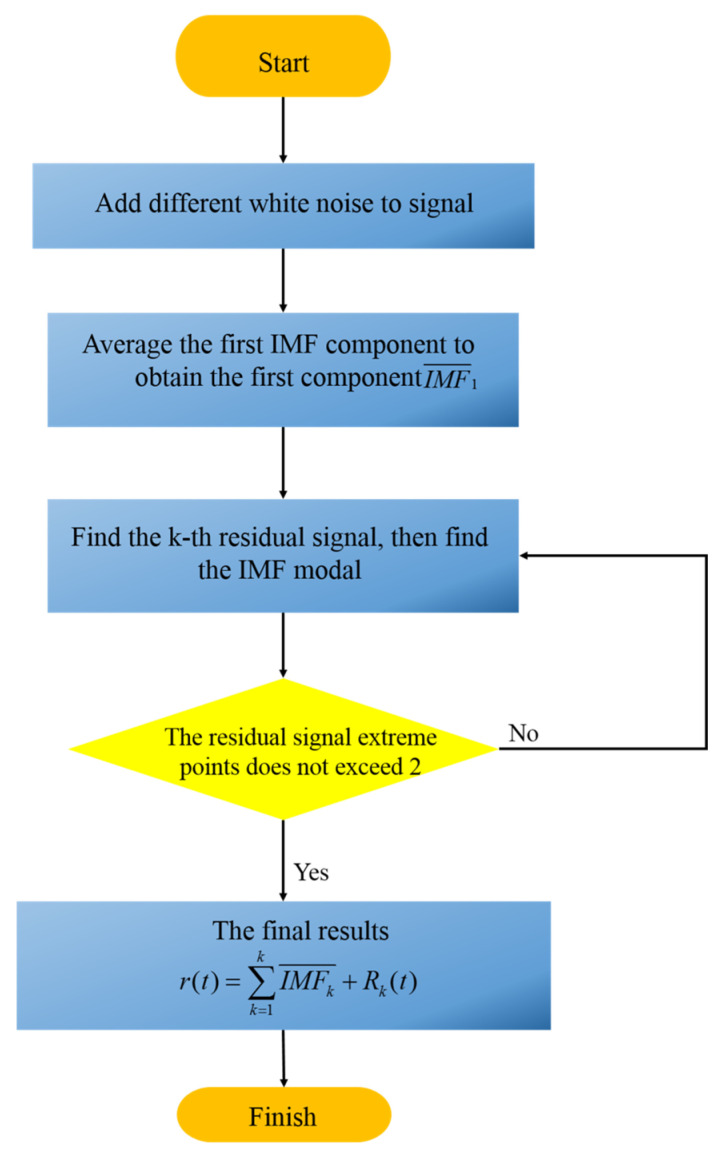
Process of CEEMDAN method.

This study proposes a novel method named CEEMDAN on the basis of EEMD. Breaking down the added white noise using the EMD method adaptively instead of adding each time can reduce the residual noise of the reconstructed signal. Figure 1 shows the steps of the CEEMDAN method.

Step 1: It is assumed that *r(t)* is the original signal, the $k\;\left(k=1,2,\ldots k\right)$ obtained by decomposing is defined as IMF and represented by ${\bar{IMF}}_{k}$, $E_{k}(\cdot )$ is the *k*th modal component obtained by EMD method, and $n_{i}$(i = 1,2,…,i) is different white noise.

Step 2: The original signal r(t) is decomposed, and then the first IMF component is averaged to obtain the first IMF component ${\bar{IMF}}_{1}$. r(t) and ${\bar{IMF}}_{1}$ are shown in Equations (1) and (2):

(1) $$r_{1}(t)=r(t)+n_{i}(t)$$

(2) $${\bar{IMF}}_{1}=\frac{1}{I}\sum_{i=1}^{I}IMF_{i1}$$

Step 3: The signal $r_{1}(t)+\varepsilon E_{1}(n_{i}(t))$ is decomposed with ε representing adaptive coefficient, and the second component ${\bar{IMF}}_{2}$ is obtained in Equation (3).

(3) $${\bar{IMF}}_{2}=\frac{1}{I}\sum_{i=1}^{I}E_{1}(r_{1}(t)+\varepsilon E_{1}(n_{i}(t)))$$

Step 4: For *k* = 2, 3,……k, the *k*-th residual signal $r_{k}(t)$ is observed in Equation (4), and then the k+1 IMF modal component $\bar{IMF_{k+1}}$ is obtained as presented in Equation (5).

(4) $$r_{k}(t)=r_{k-1}(t)-\bar{IMF_{k}}$$

(5) $$\bar{IMF_{k+1}}=\frac{1}{I}\sum_{i=1}^{I}E_{1}(r_{k}(t)+\varepsilon E_{k}(n_{i}(t)))$$

The above steps are performed until the signal cannot meet the EMD decomposition conditions. The final residual signal is $R_{k}(t)$, and the original signal is expressed as Equation (6).

(6) $$r(t)=\sum_{k=1}^{k}\bar{IMF_{k}}+R_{k}(t)$$

Multi-scale arrangement entropy refers to the permutation entropy at multiple scale, the same as the arranged entropy, and can also reflect complexity and randomness of the signal. The calculation method of multi-scale arrangement entropy concentrates on using the time sequence $X=\left\{x_{1},x_{2},x_{3},\ldots ,x_{N}\right\}$ of length N to obtain its arrangement entropy after coarse graining. The specific calculation steps are as follows:

Step 1: Time sequence $X=\left\{x_{1},x_{2},x_{3},\ldots ,x_{N}\right\}$ is treated with rough granulation to obtain Equation (7).

(7) $$y_{j^{\left(s\right)}}=\frac{1}{s}\sum_{i=(j-1)s+1}^{js}x_{i}$$

where j=1,2,…,[N/s], N/s is to take [N/s] down and to reorganize, and s is a scale factor.

Step 2: The sequence reconstruction is obtained after coarseization as shown in Equation (8).

(8) $$Y_{l^{\left(s\right)}}=\left\{y_{l^{\left(s\right)}},y_{{\left(l+l\right)}^{\left(s\right)}},\ldots ,y_{{\left(l+(m-l)\lambda \right)}^{\left(s\right)}}\right\}$$

where m is an embedded dimension, λ represents delay time, and l is a reconstruction component with l=1,2,…,N−(m−1)λ.

Step 3: Liter sequence in Equation (6) is eliminated, and each coarse granular sequence can obtain a new set of sequences $s(v)=(l_{1},l_{2},\ldots ,l_{m})$ with v=1,2,…,V(V≤m!). The number of s(v) is consistent with the number of reconstruction sequences m!.

Step 4: The arrangement entropy is calculated at different scales as listed in Equation (9).

(9) $$H_{P}(m)=-\sum_{v=1}^{V}P_{v}\ln P_{v}$$

where $P_{v}$ is the probability of appearing in the v symbol sequence. It can be concluded from Equation (9) that, $P_{v}=\frac{1}{m!}$, a normalization $H_{p}(m)$, is available when $H_{p}(m)$ takes the maximum value ln(m!) as shown in Equation (10).

(10) $$H_{P}=\frac{H_{P}(m)}{\ln(m!)}$$

The time sequence appears more orderly when the value of $H_{P}$ decreases; moreover, it is more likely to be at this time. The regularity of the time sequence becomes weaker if the value of the time sequence increases, and the possibility of normal state is enhanced.

When calculating the MPE value of the signal, both the value of the embedded dimension m and the scale factor s have a significant impact on the calculation result. The range of m usually ranges from 3 to 7. When the value of m is too small, the state contains less in the reconstruction sequence, the mutant detection of the signal reduces, and the effectiveness of the algorithm declines. Large reaction to the subtle changes with the time sequence is not obvious. When s is too small, the characteristic information contained in the signal cannot be extracted effectively. To the contrary, when the value of s is too large, the complex relationship between signals may be ignored. Moreover, delay time t and data length n also have a certain influence on the analysis of time sequences [24,25]. 

### 2.2. GWO-LSSVM Algorithm

**Figure 2:**
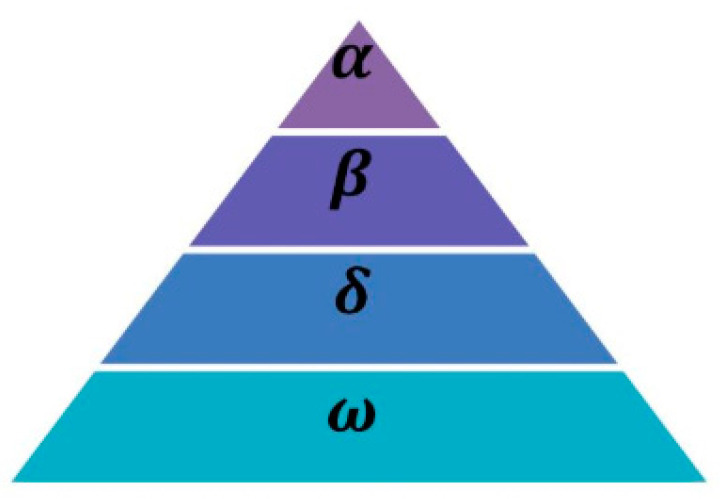
Hierarchical structure of wolf pack.

The grey wolf algorithm is proposed by learning the hunting behavior in the life of the grey wolf pack. The algorithm provides good global detection in the solution space with trivial parameters and easy implementation. The algorithm is established by first randomly initializing the population individuals to be optimized. It is necessary to calculate the fitness of each individual; depending on the difference in fitness, the three bodies with the best fitness are labeled as α wolf, β wolves, and δ wolves, and the remaining individuals are labeled as ω wolves. This is designed to imitate the strict social domination relationship with the wolves, as is shown in Figure 2. Specifically, the first layer is the α wolf, which is the leader of the pack, mainly responsible for decision-making, and is also the most outstanding wolf in the pack. The middle layer is the β wolf, which is constrained by the α wolf and helps the α wolf manage the pack. After the α wolf’s performance declines, it can be the first α wolf candidate. Due to the fact that the δ wolf exists in the middle layer of the pack, it obeys the α wolf and the β wolf, while managing the remaining wolves. Finally, the ω wolf obeys all the upper wolves in the pack. During the location update of the pack, other wolves update their positions according to the location of the optimal wolves on each layer [26].

**Figure 3:**
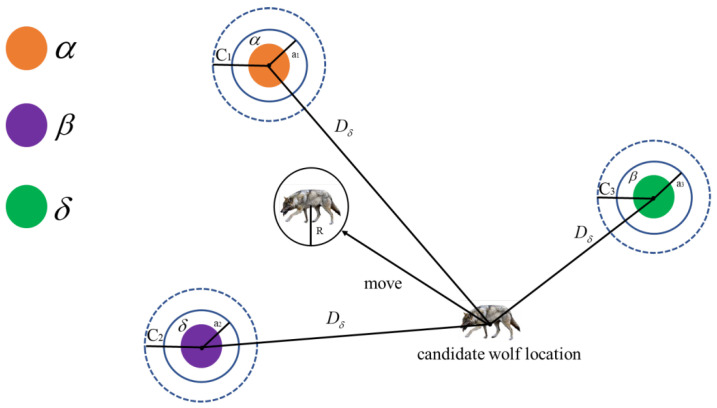
Schematic diagram of the grey wolf population optimization process.

Figure 3 shows that the optimal solution candidate positions in the entire solution space finally fall within the random circle positions defined by α, β, and δ individuals. Overall, α, β, and δ individuals need to first predict the approximate location of the optimal solution, and then the other individuals in the population update their nearby positions under the guidance of the current optimal three individuals, to complete the search for the optimal solution.

LSSVM was proposed by Suykens on the basis of SVM. It addresses the drawback that the conventional SVM leads to excessive computation when solving practical problems and realizes the inequality constraint problem of the conventional SVM into the equation constraint problem. In terms of the loss function, the conventional SVM adopts a quadratic programming method, while the LSSVM adopts a least-squares linear system. The specific form is to map the nonlinear estimation functions to a high-dimensional space using the largest number of decision-making functions to finally realize the transformation from the nonlinear functions to the linear functions.

(11) $$f(x_{i})=w^{T}•w+b,i=1,2,\ldots ,N$$

where $x_{i}$ represents the actual input data, $f(x_{i})$ represents the output label, N represents the number of samples, $w^{T}$ represents the regression coefficient, and b represents the deviation. For the regression problem of the least squares support vector machine, the optimization model is expressed in Equations (12) and (13).

(12) $$\underset{w,\xi ,b}{\min}J(w,\xi )=\frac{1}{2}w•w+\frac{\gamma }{2}\sum_{i=1}^{n}\xi_{i}^{2}$$

(13) $$s.t.y_{i}=w^{T}•\varphi (x_{i})+b+\zeta_{i},\gamma \geq 0$$

where γ is the penalty coefficient, $\zeta_{i}$ is the slack variable, and $\varphi (x_{i})$ is the mapping function. The Lagrangian function of the construction is listed in Equation (14).

(14) $$L(w,b,\xi_{i},a_{i})=J(w,\xi_{i})-\sum_{i=1}^{n}a_{i}\left\{w^{T}\varphi (x_{i})+b+\xi_{i}-y_{i}\right\}$$

where $a_{i}$ is the Lagrange multiplier, and the Lagrangian function is used to derive the derivation $w,b,\xi_{i},a_{i}$, respectively. The optimality condition of the KKT point is expressed in Equations (15)–(18).

(15) $$\frac{\partial L}{\partial w}=0\Rightarrow w=\sum_{k=1}^{N}a_{k}\varphi (x_{i})$$

(16) $$\frac{\partial L}{\partial b}=0\Rightarrow \sum_{i=1}^{N}a_{i}=0$$

(17) $$\frac{\partial L}{\partial \xi_{i}}=0\Rightarrow a_{i}=\gamma \xi_{i}$$

(18) $$\frac{\partial L}{\partial w}=0\Rightarrow w^{T}\varphi (x_{i})+b+\xi_{i}-y_{i}$$

After eliminating $\xi_{i}$ and w, Equation (19) is obtained.

(19) $$\left[\begin{array}{cc} 0 & 1^{T}\\ 1 & \Omega +\gamma^{-1}•I \end{array}][\begin{array}{c} b\\ a \end{array}]=[\begin{array}{c} 0\\ y \end{array}\right]$$

The final prediction model is shown in Equation (20).

(20) $$f(x)=\sum_{i=1}^{n}a_{i}K(x,x_{i})+b$$

where $K(x,x_{i})$ is the kernel function. This study chooses the RBF radial basis function as the kernel function, as shown in Equation (21).

(21) $$K(x,x_{i})=\exp(-\frac{{\left|x-x_{i}\right|}^{2}}{2\sigma^{2}})$$

where $x-x_{i}=\sqrt{\sum_{k=1}^{n}{\left(x^{k}-x_{i}^{k}\right)}^{2}}$, and σ is the kernel width.

**Figure 4:**
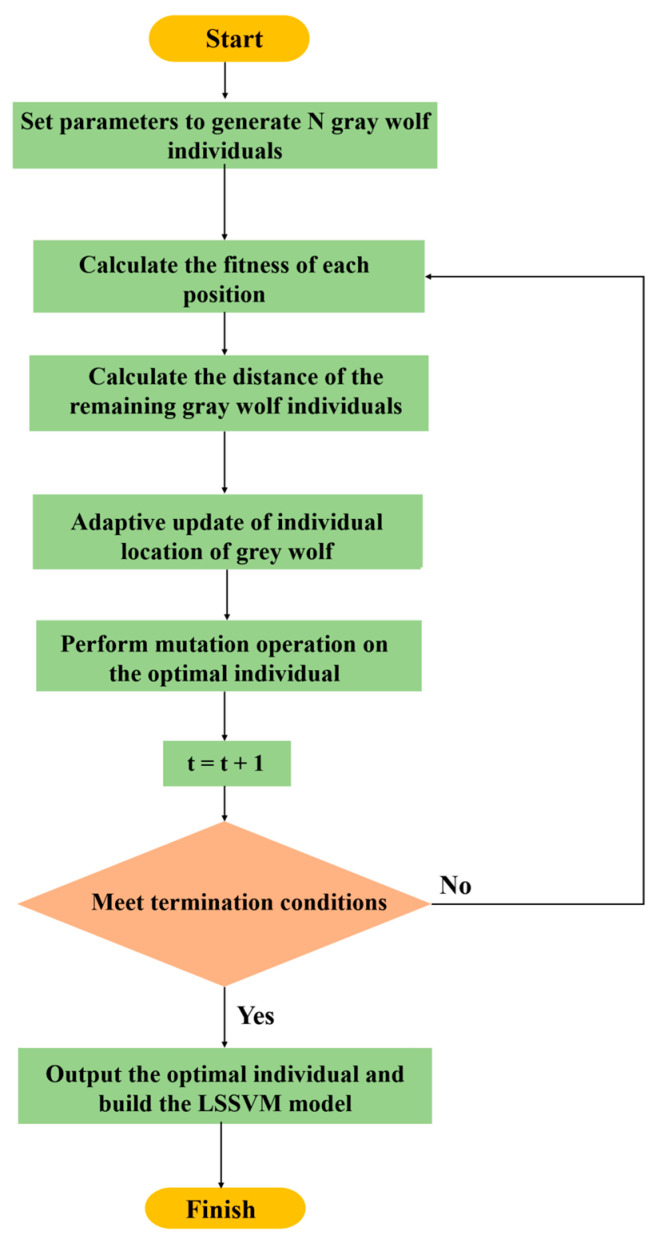
GWO-LSSVM algorithm processing flow.

In this section, it is concluded that the values of the penalty coefficient γ and the kernel parameter σ both determine the accuracy of LSSVM, but the majority of the LSSVM parameters selected in existing studies only rely on the manual experience and fail to achieve adaptive optimization, hindering its learning and generalization, and transformation ability. In the GWO-LSSVM algorithm proposed in this study, GWO is applied to optimize the two hyperparameters of LSSVM. GWO-LSSVM combines the high robustness of GWO with the low complexity of LSSVM, while achieving parameter adaptation. After reaching the maximum allowed number of iterations, LSSVM will achieve the optimal solution of the hyperparameters and achieve the purpose of optimization. The GWO-LSSVM algorithm processing flow is shown in Figure 4 [27].

Step 1: According to the IMF components obtained by CEEMDAN, the corresponding partial components and each predicted component can be found.

Step 2: The size of the grey wolf population, the maximum number of iterations, the optimization parameters, and their upper and lower bounds are initialized. The fitness function is determined, and the initial position of the individual wolf group is randomly generated.

Step 3: The individual fitness value of the wolf pack is calculated according to the determined fitness function.

Step 4: The individual wolves are sorted according to the fitness value to select the top three individuals as *α* wolf, *β* wolf, and *δ* wolf, respectively, and the remaining wolf is *ω*.

Step 5: By moving the wolf pack according to Equation (10) and to Equation (16), the position of the wolf pack is updated.

Step 6: It should be returned to Step 3 if the maximum number of iterations or accuracy requirements is met.

Step 7: The position coordinates of the α wolf are output as input parameters when using LSSVM to predict each component.

Step 8: The predicted components are integrated to determine the final prediction result.

### 2.3. Adaptive Kalman Filtering

In the actual application of FOG noise reduction, when the number of measured values k increases continuously, the deviation between the estimated value and the actual value becomes larger and larger, causing KF to gradually lose its effect and lead to the gradual failure of KF. Since Kalman is a recursive process where the number of filtering steps increases, the round error gradually accumulates, resulting in the estimated mean square error matrix being non-negative or even losing symmetry in the urban area, so that the calculated value of the gain matrix gradually loses the appropriate weighting effect, leading to divergence. Especially when the KF is applied to the FOG, the accuracy of this phenomenon will decline, and the random error will increase, which affects the use of the MEMS gyroscope. After the introduction of adaptive KF, in the filtering process, the correction of the fresh measurement value on the estimated value reduces, and the correction effect of the old measurement value increases relatively. By improving the KF equation, the concept of calibration factor is proposed, where the weight of the old measurement value gradually reduces, and the weight of the fresh measurement value increases accordingly. The divergence of the classical KF is restrained, thus reducing the randomness and error of the FOG, and meanwhile improving the accuracy of the FOG. The predicted mean square error of the originally designed KF is changed to Equation (22).

(22) $$P_{k,k-1}^{*}=\Phi_{k,k-1}(P_{k-1}^{*}•s)\Phi_{k,k-1}^{T}+\Gamma_{k-1}Q_{}\Gamma_{k-1}^{T}$$

Compared with the original KF, there is an additional calibration factor s in the formula to predict the mean square error. If *s* > 1, the total ratio $P_{k,k-1}$ is larger than $P_{k,k-1}$. Since $K_{k}^{*}=P_{k.k-1}^{*}H_{k}^{T}{\left[H_{k}P_{k.k-1}^{*}H_{k}^{T}+R_{k}\right]}^{-1}$, there is always $K_{k}^{*}>K_{k}$, indicating that this filtering algorithm is used to design the KF using the new measurement, which is more weighted than the general KF. And because ${\hat{X}}_{k}^{*}=(I-K_{k}^{*}H_{k})\Phi_{k,k-1}{\hat{X}}_{k-1}^{*}+K_{k}^{*}Z_{k}=(I-K_{k}^{*}H_{k}){\hat{X}}_{k/k-1}^{*}+K_{k}^{*}Z_{k}$,$K_{k}^{*}>K_{k}$ means that the utilization weight of ${\hat{X}}_{k/k-1}^{*}$ relatively reduces, that is, the influence of the old measured value on the estimated value decreases. The calibration factor s should be selected based on the actual engineering needs, and the best calibration factor s should be determined on the design of the adaptive KF [28,29].

## 3. Algorithm Improvement

An improved MPE-CEEMDAN method of temperature compensation is introduced in this study based on AKF and GWO-LSSVM for the FOG with the specific steps shown as follows: 

Step 1: The output data of the FOG are decomposed by the method of CEEMDAN to obtain multiple IMFs.

Step 2: The sample entropy is obtained by step 1, and the noise of the fiber gyroscope is divided into four categories by obtaining different sample entropies, namely constant noise, white noise, colored noise, and temperature error.

Step 3: Constant noise is abandoned, and the white and colored noise are sent to the adaptive KF for processing.

Step 4: The method of GWO-LSSVM is utilized to process temperature error, and the temperature error of the fiber gyroscope is further processed by establishing a temperature compensation model.

**Figure 5:**
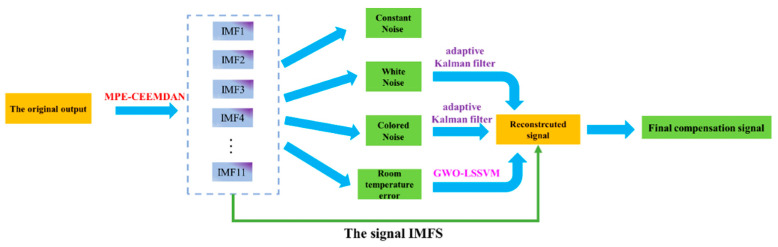
Improved algorithm based the fiber optic gyroscope for the output with temperature drift.

Step 5: The data obtained by processing step 2, step 3, step 4, and the signal IMFs are reconstructed, and the optimized optical fiber gyroscope output signal is obtained. The specific block diagram of the algorithm is shown in Figure 5.

## 4. Experiment and Analysis

### 4.1. The Experiment of FOG

**Figure 6:**
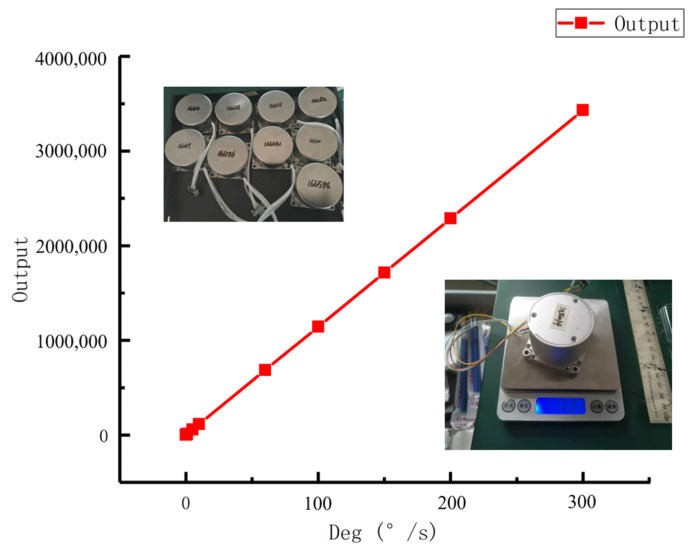
#1850014 FOG and scale factor curve.

There are some FOGs that can be used for experiments, of which the #1850014 FOG is chosen as the research objective of the experiment discussed in this chapter. The FOG and the scale factor curve are illustrated in Figure 6 and Table 1.

### 4.2. All-Range Temperature Experiment of FOG

**Figure 7:**
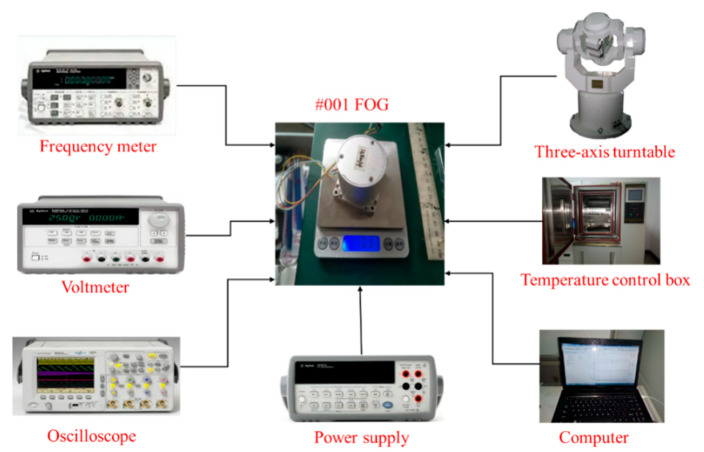
Experimental environment of FOG.

The FOG is placed in a temperature-controlled oven, and the FOG is enabled to be stabled. After the FOG is powered on and stabilized, the output data are collected. The experimental temperature environment based on #1850014 FOG is shown in Figure 7. It can be easily found that the output data at constant temperature do not show good performance. Therefore, attention should be paid to improving the performance of FOG [30,31,32].

**Figure 8:**
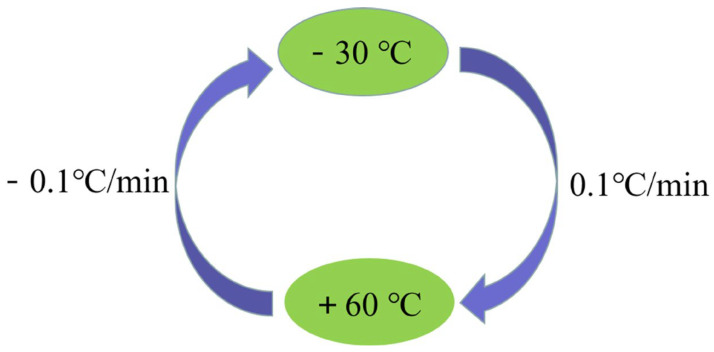
Process of all-range temperature experiment.

**Figure 9:**
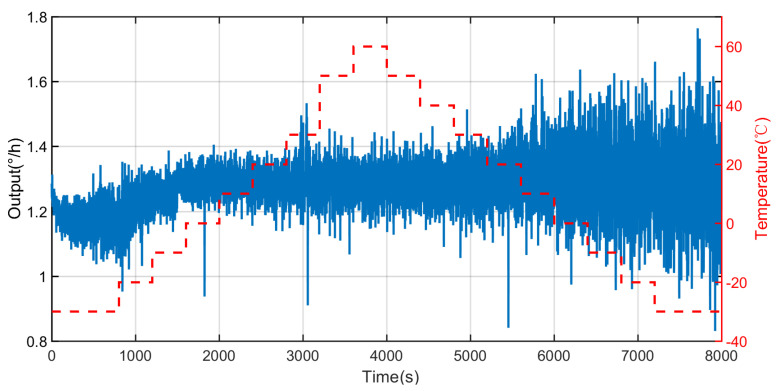
Original output of FOG based on all-range temperature experiment.

Next, the all-range temperature experiment is conducted with the following steps. The FOG is placed in the temperature-controlled oven to output the signal of the FOG. The range of temperature is set from −30 °C to 60 °C, and temperature rate is set as 0.1 °C/min. At first, the temperature is set at 20 °C and maintained for two hours to ensure stable structural temperature. Secondly, the temperature rate is raised to 0.1 °C/min. Next, the temperature of the temperature-controlled oven is increased to 60 °C. Then, the temperature is reduced from 60 °C to −30 °C at the rate of 0.1 °C/min. The process of the all-range temperature experiment is shown in Figure 8. Figure 9 displays the dramatic change in the output data in this temperature range. Therefore, more attention needs to be paid to the temperature range from −30 °C to 60 °C.

### 4.3. Data Analysis and Discussion

The temperature experiments are carried out to obtain the output data of the FOG. It can be concluded from Figure 9 that the output data of the FOG indicates a significant amount of noise and the temperature drift of the FOG is very large. 

**Figure 10:**
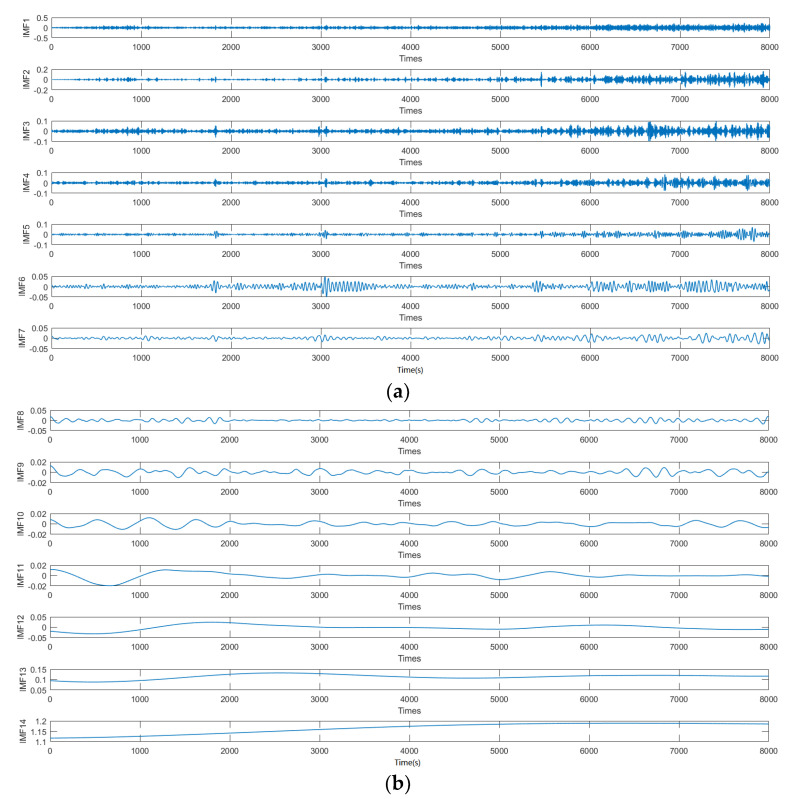
CEEMDAN decomposition results: (**a**) IMF1-IMF7 and (**b**) IMF8-IMF14.

Due to the fact that the output of the FOG is a nonlinear sequence, traditional filtering methods such as KF and wavelet threshold denoise cannot be filtered using the output of the FOG alone. Therefore, the method introduced in this study can be used to address the temperature drift and error brought by the FOG. The signal is reconstructed to the optical fiber gyroscope output by establishing a temperature compensation model. The method of CEEMDAN decomposes the output data of the FOG and obtains a total of 14 IMF components, as shown in Figure 10, which indicates that the problem of the IMF component modulus after CEEMDAN decomposed is well suppressed. The high-frequency discontinuous signals are submerged by the noise, which decomposes the low frequency useful signal very well and achieves the purpose of decomposition.

**Figure 11:**
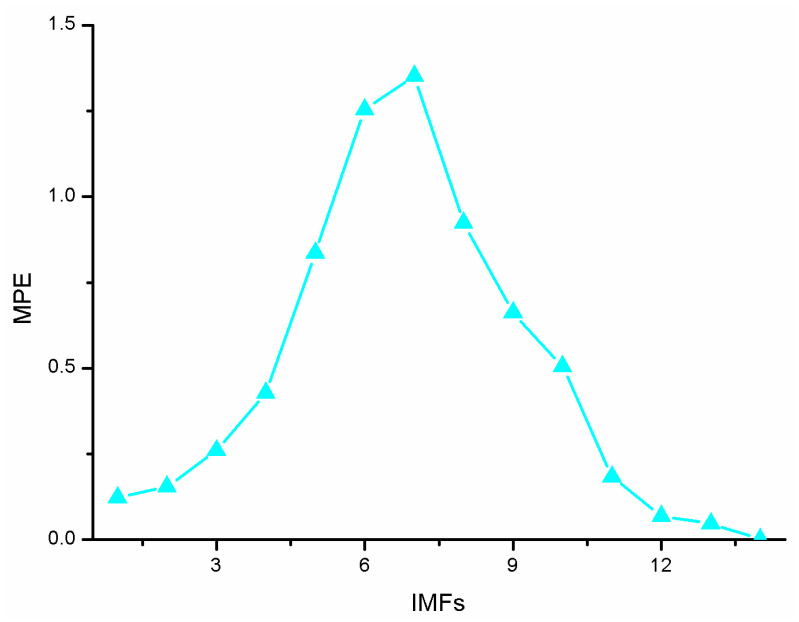
Multiscale entropy of each IMF order.

Combined with the multi-scale entropy value diagram in Figure 11, at the IMF4, the MPE increases significantly and begins to have signal components. At the IMF10, the entropy value decreases significantly, indicating that the IMF is the signal lead. The multi-scale entropy value of IMF1-IMF4 is less than 0.5, which is considered to be the IMF component dominated by noise, that is, K is 4. The multi-scale entropy value of IMF5-IMF10 is greater than 0.5, which is a mixed IMF of noise signal, that is, L is 10, and IMF11-IMF14 is a signal-dominated IMF.

**Figure 12:**
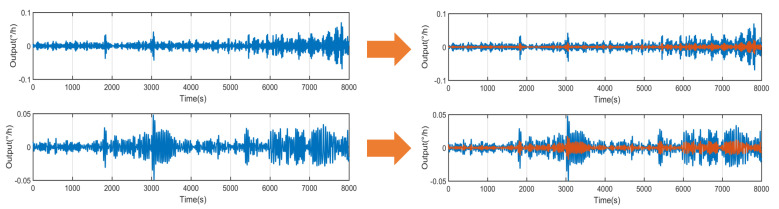
Conventional KF results.

**Figure 13:**
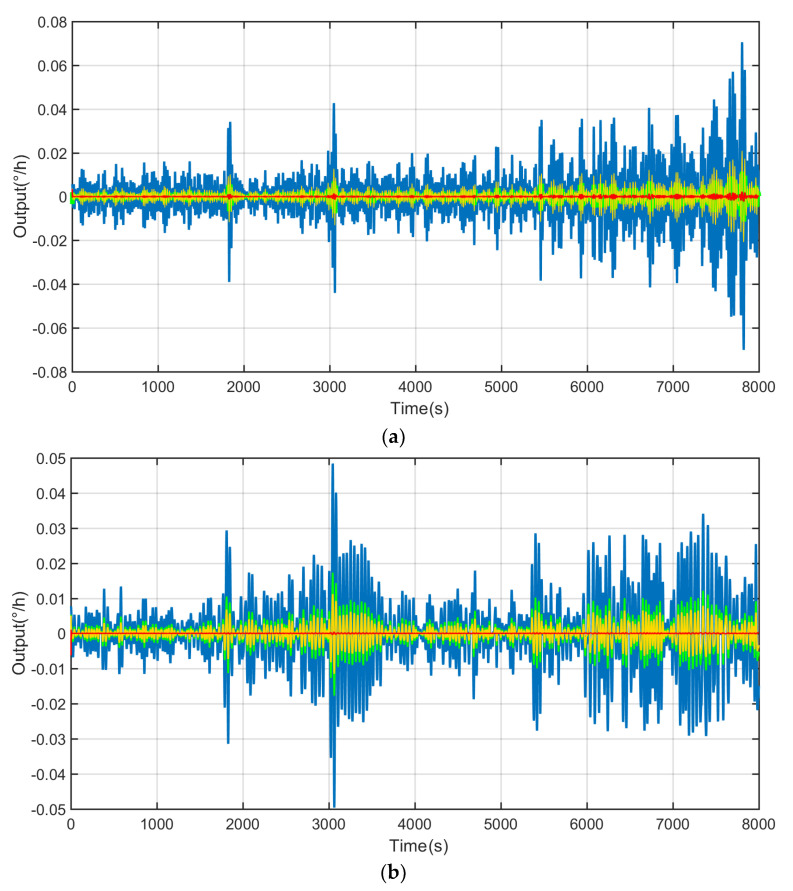
AKF results: (**a**) white noise and (**b**) color noise.

The AKF method is used to process mixed noise, which contains colored noise and white noise. The conventional KF method is used to process the mixed noise. During the filtering process, the corrective effect of the fresh quantity measurement value on the estimation value decreases, and the correction effect of the old measurement value increases relatively, as shown in Figure 12. Therefore, according to the characteristics of the mixed noise decomposed by the CEEMDAN method, the best calibration factor S is found through multiple repeated experiments, that is, S = 0.583. By using the method of AKF, the filter effect is obviously improved, as shown in Figure 13. When S = 0.583, AKF presents the best filtering effect on mixed noise (Figure 13).

**Figure 14:**
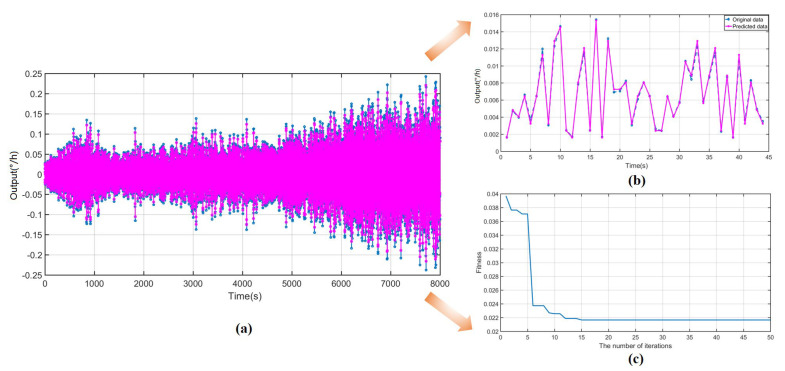
Temperature compensation model based on GWO-LSSVM: (**a**) algorithm operation results of the temperature compensation model, (**b**) specific algorithm operation results within 45 s, and (**c**) number of iterations of the algorithm.

The GWO-LSSVM combination model is used to predict the temperature drift of the fiber gyroscope, and the output and data of the fiber gyroscope are used as the learning dataset of the entire model to establish a temperature compensation model, find the temperature error, and finally eliminate the temperature error to the output of the gyroscope temperature. The number of grey wolves in the GWO optimization algorithm of the combination model is set as 50, the largest iteration number is 20, and the boundary of the parameter value is set as GAM = (10, 10^5^) and SIG2 = (10^−6^, 10^6^). The function is the equity error during the learning dataset. After using the GWO algorithm to optimize the parameters in the LSSVM algorithm, the temperature compensation model is obtained as shown in Figure 14, including the temperature error, temperature compensation model, and iteration. The temperature error compensation model of the fiber gyroscope is found to address the temperature error. The processing error of the fiber gyroscope after treatment reduces significantly to achieve good results.

**Figure 15:**
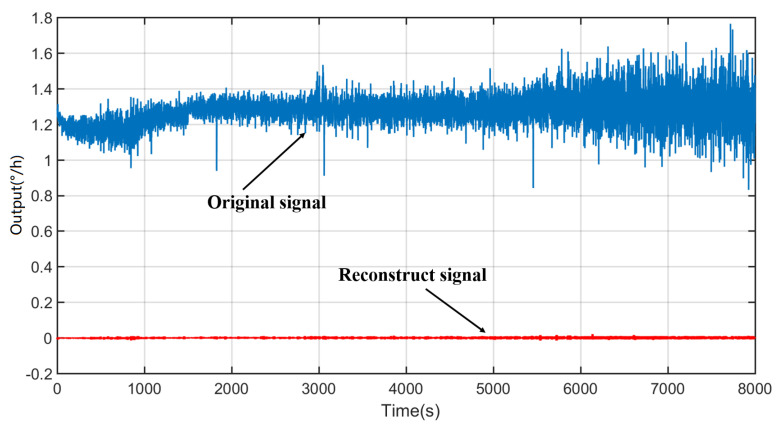
Compensation signal after reconstructing.

Subsequently, the processed aliasing noise, the processed temperature drift, and the signal-dominated IMF are reconstructed to obtain the final compensation signal, as shown in Figure 15.

**Figure 16:**
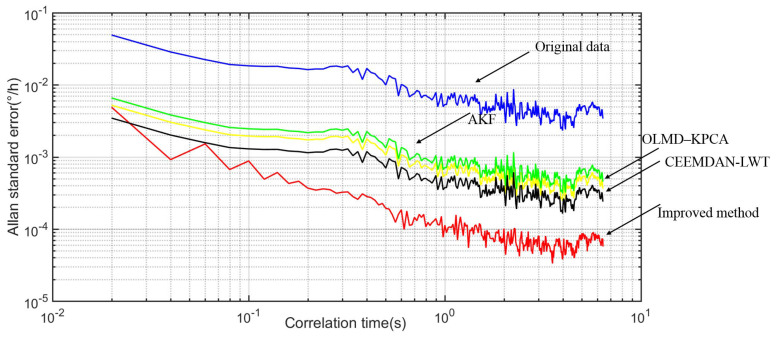
Allan variance curve comparison.

Finally, the prominent feature of Allan variance is its ease to represent and identify various sources of error and the contributions of the whole noise statistical features, and it has the advantages of easy calculation and separation. The Allan variance is widely used in FOG performance analysis as an IEEE-approved standard analysis method, as listed in Table 2. On the one hand, by using the improved method, the outputs of FOG ranged from −30 °C to 60 °C and the temperature drift reduced significantly. For example, the factor of quantization noise (Q) reduces from 6.1269 × 10^−3^ to 1.0132 × 10^−4^, the factor of bias instability (B) reduces from 1.53 × 10^−2^ to 1 × 10^−3^, and the factor of random walk of angular velocity (N) reduces from 7.8034 × 10^−4^ to 7.2110 × 10^−6^. Figure 16 illustrates the Allan variance curve comparison.

## 5. Conclusions

The detailed temperature error of the FOG is discussed by proposing an improved MPE-CEEMDAN method based on AKF and GWO-LSSVM. In the improved fusion method based on temperature experiments and compared experiments, the output of the FOG undergoes a process of temperature error search, establishing the temperature error compensation model, and filtering. The main findings are as follows:

(1) The improved MPE-CEEMDAN method based on AKF and GWO-LSSVM combines MPE-CEEMDAN, AKF, and GWO-LSSVM-related algorithms. The final output of the FOG decreases significantly compared with that of the Allan variance method, which indicates the good feasibility and effectiveness of the algorithms based on the novel method.

(2) Using the improved method, the temperature drift of the FOG ranging from −30 °C to 60 °C reduces significantly. For example, the factor of Q reduces from 6.1269 × 10^−3^ to 1.0132 × 10^−4^, the factor of B reduces from 1.53 × 10^−2^ to 1 × 10^−3^, and the factor of N reduces from 7.8034 × 10^−4^ to 7.2110 × 10^−6^.

(3) The experiments show that the method proposed in this study can greatly compensate the output signal of the FOG to obtain zero bias stability, zero bias instability, and angle random walking with stable effect. Meanwhile, the compensation of the improved MPE-CEEMDAN method based on AKF and GWO-LSSVM significantly improves with an evident compensation effect to provide a certain engineering application value.

## Figures and Tables

**Table 1:** The parameters of the #1850014 FOG used in this experiment.

Parameters	Value
Start Time (s)	<1 s
Size (mm)	Φ120×H39.5
Weight (g)	<800
Bandwidth (Hz)	>100
Manufacturer	BEWIS SENSING
Sensitivity (°/h)	≤0.0007
Measuring range (°/s)	−500~+500
Operation temperature (°C)	−40~+65

**Table 2:** The Allan variance of compared experiment based on original signal and the improved method.

	Original Data	AKF [22]	OLMD–KPCA [19]	CEEMDAN-LWT [33]	Improved Method
$Q({}^{\circ }$)	6.1269 × 10−4	-	1.4421 × 10−3	2.3125 × 10−4	1.0132 × 10−4
$B({}^{\circ }/h$)	1.5323 × 10−2	5.4223 × 10−4	2.1259 × 10−3	5.6311 × 10−4	1.1504 × 10−4
$N({}^{\circ }/\sqrt{h}$)	7.8034 × 10−4	1.2657 × 10−4	4.268 × 10−5	1.2956 × 10−5	7.2110 × 10−6
Time consumption (s)	-	0.5321	1.9845	3.2767	3.6234

## Data Availability

Not applicable.

## References

[1] Cao H., Xue R., Cai Q., Gao J., Zhao R., Shi Y., Huang K., Shao X., Shen C. (2020). Design and Experiment for Dual-Mass MEMS Gyroscope Sensing Closed-Loop System. IEEE Access.

[2] Cao H., Liu Y., Zhang Y., Shao X., Gao J., Huang K., Shi Y., Tang J., Shen C., Liu J. (2019). Design and Experiment of Dual-Mass MEMS Gyroscope Sense Closed System Based on Bipole Compensation Method. IEEE Access.

[3] Cao H., Li H., Shao X., Liu Z., Kou Z., Shan Y., Shi Y., Shen C., Liu J. (2018). Sensing mode coupling analysis for dual-mass MEMS gyroscope and bandwidth expansion within wide-temperature range. Mech. Syst. Signal Process..

[4] Shen C., Li J., Zhang X., Tang J., Cao H., Liu J. (2016). Multi-scale parallel temperature error processing for dual-mass MEMS gyroscope. Sens. Actuators A Phys..

[5] Shen C., Song R., Li J., Zhan X., Tang J., Shi Y., Liu J., Cao H. (2015). Temperature drift modeling of MEMS gyroscope based on genetic-Elman neural network. Mech. Syst. Signal Process..

[6] Xia D., Cheng Y., Kong L. (2014). The Development of Micromachined Gyroscope Structure and Circuitry Technology. Sensors.

[7] Gao W., Wang Z., Wang G., Miao W. (2019). Angular Random Walk Improvement of Resonator Fiber Optic Gyro by Optimizing Modulation Frequency. IEEE Photonics J..

[8] Yang G., Liu Y., Li M., Song S. (2015). AMA- and RWE- Based Adaptive Kalman Filter for Denoising Fiber Optic Gyroscope Drift Signal. Sensors.

[9] Gao Y., Guan L., Wang T., Sun Y. (2015). A Novel Artificial Fish Swarm Algorithm for Recalibration of Fiber Optic Gyroscope Error Parameters. Sensors.

[10] Wang W., Chen X. (2016). Temperature drift modeling and compensation of fiber optical gyroscope based on improved support vector machine and particle swarm optimization algorithms. Appl. Opt..

[11] Shen C., Cao H., Li J., Tang J., Zhang X., Shi Y., Yang W., Liu J. (2016). Hybrid de-noising approach for fiber optic gyroscopes combining improved empirical mode decomposition and forward linear prediction algorithms. Rev. Sci. Instrum..

[12] Wang P., Li G., Gao Y. (2022). A compensation method for gyroscope random drift based on unscented Kalman filter and support vector regression optimized by adaptive beetle antennae search algorithm. Appl. Intell..

[13] Zhang W., Zhang D., Zhang P., Han L. (2022). A New Fusion Fault Diagnosis Method for Fiber Optic Gyroscopes. Sensors.

[14] Zhao S., Guo C., Ke C., Zhou Y., Shu X. (2022). Temperature drift compensation of fiber strapdown inertial navigation system based on GSA-SVR. Measurement.

[15] Cao Y., Xu W., Lin B., Zhu Y., Meng F., Zhao X., Ding J., Li Z., Xu Z., Yu Q. (2022). A method for temperature error compensation in fiber-optic gyroscope based on machine learning. Optik.

[16] Brzostowski K., Swiatek J. Empirical Mode Decomposition Based Denoising Algorithm for Fiber Optical Gyroscope Measurement. Proceedings of the 25th International Conference on Systems Engineering.

[17] Wang W., Chen X. (2018). Multiscale modeling of fiber optic gyroscope temperature drift based on improved ensemble empirical mode decomposition. Appl. Opt..

[18] Wang P., Gao Y., Wu M., Zhang F., Li G., Qin C. (2020). A Denoising Method for Fiber Optic Gyroscope Based on Variational Mode Decomposition and Beetle Swarm Antenna Search Algorithm. Entropy.

[19] Song R., Chen X. (2017). Analysis of fiber optic gyroscope vibration error based on improved local mean decomposition and kernel principal component analysis. Appl. Opt..

[20] Zhang X., Cao H., Shao X., Liu J., Shen C. (2018). FOG De-Noising Algorithm Based on Augmented Nonlinear Differentiator and Singular Spectrum Analysis. Appl. Sci..

[21] Wang D., Xu X., Zhang T., Zhu Y., Tong J. (2019). An EMD-MRLS de-noising method for fiber optic gyro Signal. Optik.

[22] Song N., Yuan Z., Pan X. (2019). Adaptive Kalman filter based on random-weighting estimation for denoising the fiber-optic gyroscope drift signal. Appl. Opt..

[23] Liu C., Yang Z., Shi Z., Ma J., Cao J. (2019). A Gyroscope Signal Denoising Method Based on Empirical Mode Decomposition and Signal Reconstruction. Sensors.

[24] Shen C., Zhang Y., Guo X. (2021). Seamless GPS/Inertial Navigation System Based on Self-Learning Square-Root Cubature Kalman Filter. IEEE Trans. Ind. Electron..

[25] Shen C., Zhang Y., Tang J. (2019). Dual-optimization for a MEMS-INS/GPS system during GPS outages based on the cubature Kalman filter and neural networks. Mech. Syst. Signal Process..

[26] Huang H., Tang J., Song R., Tang X. (2022). A novel matrix block algorithm based on cubature transformation fusing variational Bayesian scheme for position estimation applied to MEMS navigation system. Mech. Syst. Signal Process..

[27] Huang H., Wei J., Wang D., Zhang L., Wang B. (2022). In-Motion Initial Alignment Method Based on Vector Observation and Truncated Vectorized K-matrix for SINS. IEEE Trans. Instrum. Meas..

[28] Torres M.E., Colominas M.A., Schlotthauer G., Flandrin P. (2011). A Complete Ensemble Empirical Mode Decomposition with Adaptive Noise. Proceedings of the 2011 IEEE International Conference on Acoustics, Speech and Signal Processing (ICASSP), ICASSP-11.

[29] Saxena M.K., Raju S.J., Arya R., Pachori R.B., Ravindranath S.V.G., Kher S., Oak S.M. (2016). Empirical mode decomposition-based detection of bend-induced error and its correction in a Raman optical fiber distributed temperature sensor. IEEE Sens. J..

[30] Narasimhappa M., Nayak J., Terra M.H., Sabat S.L. (2016). ARMA model based adaptive unscented fading Kalman filter for reducing drift of fiber optic gyroscope. Sens. Actuator A Phys..

[31] Narasimhappa M., Sabat S.L., Nayak J. (2016). Fiber-Optic Gyroscope Signal Denoising Using an Adaptive Robust Kalman Filter. IEEE Sens. J..

[32] Komaty A., Boudraa A., Augier B., Dareemzivat D. (2014). EMD-Based Filtering Using Similarity Measure Between Probability Density Functions of IMFs. IEEE Trans. Instrum. Meas..

[33] Dang S.-W., Li L.-J., Wang Q.-Q., Wang K.L., Cheng P.-Z. (2020). Fiber optic gyro noise reduction based on hybrid CEEMDAN-LWT method. Measurement.

